# Keeping the Breath in Mind: Respiration, Neural Oscillations, and the Free Energy Principle

**DOI:** 10.3389/fnins.2021.647579

**Published:** 2021-06-29

**Authors:** Asena Boyadzhieva, Ezgi Kayhan

**Affiliations:** ^1^Department of Philosophy, University of Vienna, Vienna, Austria; ^2^Department of Developmental Psychology, University of Potsdam, Potsdam, Germany; ^3^Max Planck Institute for Human Cognitive and Brain Sciences, Leipzig, Germany

**Keywords:** interoception, respiration-entrained neural oscillations, controlled breathing, free-energy principle, self-regulation

## Abstract

Scientific interest in the brain and body interactions has been surging in recent years. One fundamental yet underexplored aspect of brain and body interactions is the link between the respiratory and the nervous systems. In this article, we give an overview of the emerging literature on how respiration modulates neural, cognitive and emotional processes. Moreover, we present a perspective linking respiration to the free-energy principle. We frame volitional modulation of the breath as an active inference mechanism in which sensory evidence is recontextualized to alter interoceptive models. We further propose that respiration-entrained gamma oscillations may reflect the propagation of prediction errors from the sensory level up to cortical regions in order to alter higher level predictions. Accordingly, controlled breathing emerges as an easily accessible tool for emotional, cognitive, and physiological regulation.

## Introduction

“The “I think” which Kant said must be able to accompany all my objects, is the “I breathe” which actually does accompany them. Breath is the essence out of which philosophers have constructed the entity known to them as consciousness” (James, 1912).

In Sanskrit “prana” means “breath.” At the same time it means “life.” The word “atman” is similarly translated as both soul and air. This linguistic tie is a global one, present in many ancient cultures: Ancient Greek has the concepts of “psyché” and “pneuma,” in Hebrew there is “ruach” and “néfesh,” as well as the Latin “spiritus” and “anemos,” the Chinese “ch’i,” Japanese “ki” and Arabic “ruh.” The reason is straightforward - breath brings us into life and carries us all the way through it. Some cultures have recognized its importance thousands of years ago, adopting various breathing exercises as a part of their spiritual practices. Interest in these practices has since spread from the East to the West and from the spiritual to the scientific. While research on whether respiration influences our cognitive, emotional and physiological functioning is on the rise, surprisingly little is known about the underlying mechanisms of how respiration modulates these processes. In this article, we review the state-of-the-art understanding of how respiration interacts with a wide network of both central and peripheral systems, which work together to maintain homeostasis. We provide a systematic overview of the emerging literature on how respiration modulates neural, cognitive and emotional processes. Moreover, we present an outlook for future work on formalizing volitional modulation of breath as an *active inference* mechanism.

While increasing the awareness of an inner sensory modality would be a step to modulating it, not every interoceptive signal is an optimal candidate. The heartbeat, for instance, has been well-studied for its affective and cognitive interrelationships (Garfinkel et al., 2016; Park and Blanke, 2019). Nevertheless, unless excited, heart rhythm remains on the periphery of experience for most of the people, and even more importantly, cannot be directly modulated. The breath, however, is unique. Despite being an interoceptive modality, it can be easily brought into awareness, and its tempo can be controlled.

Consider the following example: Sam, a graduate student of psychology, is attending a lecture on artificial intelligence. While listening to the speaker, a question outside of their expertise arises. While planning on how to formulate the question, Sam’s heart starts racing. Sam is anxious. This realization alone does not slow down the heartbeat. Sam decides to do what usually helps: to focus on the breath and slow it down. Soon after, Sam may notice that the heartbeat has normalized, and the tension has dissolved. Slowing down the breathing rhythm triggered cardiovascular, endocrine, autonomic and central brain systems, most notably the activation of the parasympathetic system, thus, the elicitation of physiological relaxation (Russo et al., 2017; Zaccaro et al., 2018). Now, Sam feels calmer to ask the question.

Linking the volitional and the autonomic, controlled breathing becomes an anchor in the present, which provides organisms with new evidence to update their beliefs on interoceptive states. This is a powerful position to be in within the free-energy framework. Through active inference, that is, changing the breathing rhythm, respiration can alter interoceptive predictions, and the felt experience thereof. As respiration-related sensory evidence is recontextualized, the body’s predictions are altered: in the case of slowing down the breathing rhythm, they become aligned with a relaxed state, rather than other, possibly conflicting, and interoceptive states (i.e., an increased heartbeat and an anxious state). This formulation, therefore, accounts for the efficacy of a slow breathing rhythm to achieve a calm emotional and physiological state (Philippot et al., 2002; Ma et al., 2017). At the same time, it acknowledges its bidirectionality, explaining how an accelerated breathing rhythm can also upregulate the system in a way that results in an aroused state (Nardi et al., 2009).

In this article, we will give an overview of the theoretical and empirical findings on how respiration affects neural, cognitive and emotional processes. Moreover, we will present an outlook for future work on how controlled breathing can be used as an active inference mechanism to modulate interoceptive predictions. Controlled breathing is proposed to be a dynamic regulatory mechanism, which is used as a means to update top-down interoceptive predictions by weighting bottom-up sensory evidence. It should be noted that here by learning to control the breath, we put emphasis on learning to actively modulate the breathing rhythm. Hence, controlled breathing is used as an umbrella term for any kind of volitional change of breathing pattern, which includes not only slowing down of the breath but also rapid breathing, as well as alterations between the two. That is, changes in rate, depth and duration of breathing can be used to both downregulate and upregulate the system eliciting distinct cognitive, affective, and physiological changes.

## Breathing Free (Energy)

### Predictive Processing and Interoceptive Models

Predictive processing has become an influential framework in understanding brain-body interactions (Seth and Friston, 2016; Barrett, 2017). According to this framework, the nervous system predicts how changes in the environment or in the body will affect the organism by creating the so-called *generative models* based on which the predictions emerge (Friston, 2010). These models don’t come as a given but they are acquired through a unique history of experience (Seth and Friston, 2016). The *predictions* issued by the nervous system are compared to the available sensory information (Friston, 2010). Whenever there is a mismatch between what is predicted and what is observed, this elicits *a prediction error* (PE), which propagates back to cortical regions (Friston, 2010). The organism then tries to reduce prediction errors either by active inference, that is, by changing its sensory states through action to update its predictions, or by changing its internal dynamics with respect to environmental conditions, conceptualized as *perceptual inference* (Friston, 2010). This error minimization process, central to the *free-energy principle*, suggests that organisms continuously try to minimize the free energy arising from discrepancies between the predicted and the observed states to eventually increase the accuracy of their internal models (Friston, 2010). In the case of interoceptive priors, the more predictable a prediction is, the less need there is to check in with the sensations felt right now (Barrett and Simmons, 2015). The reason for this is plain. If you experience your heartbeat in a predictable manner often enough, there is no need to update the priors associated with it. However, even persistent predictions, such as the interoceptive ones, are malleable (Adams et al., 2013).

*Allostasis*, the process of adaptively altering physiological parameters of the body with respect to environmental changes (Sterling, 1988), is the main objective of predictive processing within the nervous system (Sterling, 2014). Allostasis achieves *homeostasis*, which maintains physiological parameters within a relatively constant range, by ensuring that the organism adapts to a precarious environment (Sterling, 2014). The management of these parameters requires constant handling of immense amounts of information, which the nervous system achieves by employing a clever strategy: instead of processing all of the information, it only registers the discrepancies from what was expected (Friston, 2010). Thus, the brain-centered predictive regulation of allostasis coordinates changes in bodily systems to assure that the organism is prepared to meet the body’s basic needs, such as food, warmth and cooling, before they are at stake, to keep the system in equilibrium (Sterling, 2014; Kleckner et al., 2017).

Consider the example of Sam again. Instead of dwelling on the symptoms of anxiety, Sam redirects their attention to the breath. By slowing it down, Sam is taking an action to meet another internal generative model: that of being relaxed. Through active inference, mediated by controlled respiratory signals, the breath induces an almost immediate change in interoceptive priors and internal states thereof. In other words, controlled breathing emerges as a rapid and easily accessible PE minimization strategy engaging both attention and action to alter interoceptive predictions.

### Attention and Prediction Updating

Attention is a way of amplifying what is relevant and salient, leaving the rest in the background (Feldman and Friston, 2010). Usually, the regulation of breath happens without paying attention to it and the physiological and emotional changes that come with it follow automatically. However, attention can be used as a tool to deliberately control the breath. In predictive processing terms, attention weights PEs by increasing the *precision* of the sensations being attended to Smout et al. (2019). What this means is that bottom-up information, happening now, is prioritized over top-down predictions based on previous experience. Consequently, attention opens the possibility to re-evaluate sensations by amplifying their importance, which contributes to the updating of associated priors.

There are factors to consider when talking about attention in the context of controlled breathing. Redirecting the attention to the sensations of the breath can be seen as a form of interoceptive attention, which could be affected by *how* one focuses on the breath. For instance, attention can be paid to the nostrils, the rising and falling of the chest, or more subtle inner changes. Alternatively, in cases where biofeedback is used, the attention may be directed outward (Wang et al., 2010). Therefore, defining and measuring attention to the breath poses a serious experimental difficulty, which should be carefully considered in experimental designs. A good starting point would be to clarify whether the mechanisms underlying attention, as defined by the free-energy principle, can be generalized for both internal and external attention (Clark, 2017). Given that these converge in the anterior insular cortex (AIC), it is likely that both internal and external attention work together to attune the body’s internal state to the external environment (Farb et al., 2013a).

Farb et al. (2013a) operationalized an interoceptive attention task, during which participants were asked to monitor their breath without intentionally changing its rhythm. This internal attention task was contrasted to two external attention tasks. In the first external attention task, the participants were asked to “keep their minds blank” while reading sequentially presented words, that is, to suppress any cognitive or emotional response to the words. In the second task, participants pressed a button whenever they recognized a previously presented word. Using functional magnetic resonance imaging (fMRI), Farb et al. (2013a) found that interoceptive and exteroceptive attention tasks recruited different neural networks. Whereas external attention recruited frontoparietal “executive” attention network, interoceptive attention recruited primary interoceptive and somatosensory regions, as well as the posterior cingulate and hippocampus (Farb et al., 2013a). Despite engaging different attentional networks, internal and external states meet in the AIC, where internal signals may be integrated in a broader exteroceptive context (Craig, 2009; Singer et al., 2009; Farb et al., 2013a). Empirical evidence about the integration of interoceptive and exteroceptive information, however, remains scarce (Allen et al., 2019; Allen, 2020).

Expectation of a stimulus also influences the way attention contributes to changing predictions. It has been shown that precision increases the most when stimuli are predicted to occur, leading to postsynaptic gain of the PE neurons that communicate with neurons already “primed” to the stimuli (Kok et al., 2012). Perceptual inference takes advantage of this synergistic relationship between attention and prediction, which, in the context of breathing, can be related to the process of simply observing the breath. It is not clear, however, whether this is enough to minimize PEs. According to the free energy principle, to completely diminish PE, action needs to be taken (Friston, 2010). While one might argue that deliberately choosing to pay attention to something, such as the breath, is an act in its own right, the argument remains open to debate (Clark, 2017). In either case, controlled breathing can be seen as an easily accessible active inference strategy engaging both attention and action to alter interoceptive generative models.

## Breathing In: Brain-Body Interactions

Changes in the breathing rhythm are commonly associated with changes in peripheral signals mediated through the vagus nerve (Russo et al., 2017). This, however, is an incomplete view on the system-wide changes that breathing elicits. The following section reviews the state of the art demonstrating how respiration modulates sensorimotor, emotional and cognitive processes.

### Breathing in Sensation and Motion

Respiration is first and foremost a movement a rhythmic oscillation. The connection between respiration and movement is readily utilized in the martial arts, where the force of punches is maximized by synchronizing them with expirations. The tactic is justified: peak force, eye and finger-movements have all been correlated with the respiratory cycle (Rassler and Raabe, 2003; Li and Laskin, 2006; Li and Rymer, 2011).

A recent study by Park et al. (2020) demonstrated that breathing is coupled to voluntary action control. By measuring cortical readiness potential, Park et al. (2020) reported that breathing phases were temporally aligned with voluntary actions, where actions were initiated more often during the expiration period. However, it should be noted that the data presented by Park et al. (2020) is low-pass filtered in respect to the mean cycle presented. Thus, it is likely that the observed cortical readiness potential was not coupled with the periods of inspiration (I) and expiration (E) *per se*, but with the transitions between them. A closer look at the data of Park et al. (2020) shows that it is the transition from the expiration to inspiration (i.e., late phase of expiration) during which participants initiate voluntary actions more frequently. Readiness potential amplitudes were also smaller during the expiration phase as compared to inspiration. This interpretation of the results is further supported by a recent study of Dhingra et al. (2020) showing that the transitions between inspiration and post-inspiration are especially relevant for synaptic engagement: the I-PI transition exclusively engages a brainstem-wide respiratory network.

Emerging evidence further shows that the phase and depth of respiration modulate oscillatory activity in the sensorimotor cortex (Kluger and Gross, 2020). For example, in a study by Kluger and Gross (2020), magnetoencephalography (MEG) was used to study the interplay of breathing, brain activity and muscle activity. By measuring corticomuscular coherence in the beta band, Kluger and Gross (2020) showed that the rhythm of the breath, maintained by respiratory muscles, is in bi-directional interaction with brain oscillations. Importantly, the observed neural signature was dependent on whether breathing was voluntary or automatic a finding explained by the distinct neural recruitment required to meet the different demands on muscle control. Overall, their study supports the idea that controlled breathing plays a special role in mediating respiration-entrained brain synchrony enhancing motor activity (Mckay et al., 2003) and synchrony in the motor cortex (Herrero et al., 2018).

Processing of sensory signals can also be dramatically influenced by the breath (Brown and Gerbarg, 2005; Arch and Craske, 2006; Ma et al., 2017). Perhaps the most striking example has to do with pain perception, where numerous studies have shown that respiration modulates the ways in which noxious stimuli are felt or appraised (Iwabe et al., 2014; Larsen et al., 2019; Jafari et al., 2020; Wells et al., 2020). The attenuation of pain associated with slow deep breathing, especially with a low inspiration-expiration ratio, occurs independently from cardiovascular changes (Jafari et al., 2020). The endogenous opioid system, which is also activated by a slow-breathing rhythm, does not seem to be involved in the process of pain attenuation suggesting that slow deep breathing reduces pain through an independent pathway (Wells et al., 2020). While the exact mechanisms underlying how the breath modulates mobility, emotional processing, cognitive functioning, and sensory perception, are yet to be delineated, and mounting evidence suggests that large-scale synchronizations of neural rhythms are pivotal players (for review Varga and Heck, 2017).

### Breathing in Emotions

Controlled breathing can be used as an anchor into the present, which can help in the process of emotion recognition and regulation. The prime example of how breathing affects emotion is the physiological and psychological relaxation that occurs when the breathing rhythm is slowed down to 0.1 Hz or 6 breaths per minute, a technique know as slow-paced breathing (Brown and Gerbarg, 2005). This type of breathing is associated with respiratory sinus arrhythmia (RSA), a cardiorespiratory synchronization in which the interval between consecutive heartbeats decreases during inspiration and increases during expiration (Eckberg et al., 1985). Slow breathing also increases the variation in time intervals between heartbeats, or HRV - an indicator of a healthy systemic balance and ability to respond to physiological functioning (Shaffer et al., 2014). Conversely, HRV is known to be reduced by a fast breathing rhythm (Song and Lehrer, 2003). On one hand, this effect is highly relevant for stress-related disorders, such as anxiety and depression, in which HRV is known to be reduced (Gorman and Sloan, 2000). On the other hand, it shows how deeply intertwined the rhythms of the body are (Mather and Thayer, 2018). Importantly, cardiorespiratory coupling is considered to be bidirectional: cardiac signals also modulate breathing rate (Dick et al., 2014).

While breathing rapidly is often associated with fear or high arousal, it may also serve an important role in stimulating brain areas responsible for information processing in the brain, which aids responding faster to stimuli in the environment (Zelano et al., 2016). Whether this is adaptive or maladaptive, depends solely on the context. For instance, it has been shown that cyclic hyperventilation followed by breath retention activates the sympathetic nervous system and attenuates aberrant immune responses - a response appropriate for times in which an alert state is desired (Kox et al., 2014). However, in susceptible populations, panic attacks have been shown to be induced by hyperventilation (Clark, 1993). Moreover, breathing techniques which integrate aspects of both rapid and slow breathing have been shown to be effective in the treatment of stress, anxiety and depression (Brown and Gerbarg, 2005). Preliminary evidence suggests that these changes are associated with changes in emotion-related brain regions, like the amygdala, the AIC, ACC, and the prefrontal cortex (PFC) (Novaes et al., 2020). Overall, affective responses seem to be highly related to the breathing rhythm, a change which has been shown to be sufficient to induce both positive and negative emotions (Philippot et al., 2002). What these coupled variations in breathing pace and physiological responses show is that the breath can work as both an up and a down regulator of arousal. Learning to control the breath means learning to control the effects it elicits.

### Breathing in Cognition

The ups and downs of the breath are not only reflected in emotion they shape our cognitive functioning as well. And the two work hand in hand. For instance, nasal breathing phase has been shown to affect both emotional judgments and memory recall, with people being able to identify emotional faces quicker and remember an object better when presented at inhalation than exhalation (Zelano et al., 2016). In this study, intracranial electroencephalography (iEEG) recordings from epileptic patients showed how breathing is entrained with slow cortical rhythms in the olfactory cortex, which in turn modulate higher frequency rhythms in the amygdala and hippocampus - regions closely related to affective and cognitive functioning (Zelano et al., 2016). To test whether breathing phase is indeed related to emotion and memory, Zelano et al. (2016) recruited an independent group of healthy participants to test whether their recognition of emotional faces was affected by breathing phase. Results showed that the recognition of emotional faces was dependent on the respiratory phase in which the stimuli were presented. More specifically, fearful faces presented at the transitions between respiratory phases were recognized faster as participants inhaled as compared to faces presented during exhalation. This effect diminished when shifting from nasal to mouth breathing (Zelano et al., 2016). A detailed inspection of the iEEG data suggests that, in many participants, the mean spectral power increases occur as participants inhale. Similarly, the findings of Ito et al. (2014) show that increases in delta/gamma power are most reliably detected at the transition from inspiration to post-inspiration (I-PI).

While studies linking respiration to memory in humans are scarce (Huijbers et al., 2014; Arshamian et al., 2018), research done in rodents support of the role of respiratory-entrained hippocampal oscillations (Yanovsky et al., 2014; Liu et al., 2017) and their pivotal involvement in memory consolidation and memory retrieval (Kay et al., 2009; Kay, 2014). The need to further investigate how exactly the respiratory phase is linked to memory encoding and retrieval in humans remains an important line of future research.

A further link between respiration and cognition is established in a small region in the brainstem responsible for synthesizing norepinephrine (NE), the locus coeruleus (LC) (Melnychuk et al., 2018). LC, being a major player in attention and behavioral flexibility (Aston-Jones et al., 1999), is an organic link between respiration and attention (Herrero et al., 2018; Melnychuk et al., 2018). As demonstrated by Melnychuk et al. (2018) pupil dilation, a reliable indicator of LC activity (Murphy et al., 2014), has been shown to increase with inhalation and decrease during expiration. Further, Melnychuk et al. (2018) framed the respiratory-LC-attentional system as a dynamical system in which breath-focused practices can be used to influence its stability. In such a coupled dynamical system, changing one aspect of it inevitably changes the others. Therefore, by controlling the breath, one can also modulate the LC-entrained attentional system and responses relevant for the regulation of arousal, attention and stress (Berridge and Waterhouse, 2003; Yackle et al., 2017). Moreover, LC-NE activity under arousal promotes not only selective attention but also memory exemplifying the close connection between different cognitive faculties (Mather et al., 2016).

## An Embodied Neuroarchitecture

Since breathing is simultaneously an interoceptive signal and a movement within volitional control, it comes as no surprise that both the interoceptive and the proprioceptive brain networks get activated by diaphragmatic movements through sensory medullary pathways (Heck et al., 2017). Respiratory-entrained neural circuitry, however, extends beyond areas responsible for breathing regulation. This section provides a concise overview of the relevant neuroanatomy, focusing on the circuitry recruited by gamma-band frequency neural oscillations.

### Breathing in Rhythm: Neural Oscillations

Life is governed by rhythm. Brain, being a component of it, is no different. Brain functions are governed by oscillatory activity (Buzsaki, 2006). Spontaneous neural activity reflects how the brain integrates neural information on multiple spatial and temporal scales (Engel et al., 1999; Buzsaki, 2006). Emerging evidence shows that neural oscillations entrain to respiration and that respiration constitutes a fundamental neural rhythm, which is deeply intertwined with cognitive and affective functions like memory, attention and sensory processing (Zelano et al., 2016; Heck et al., 2017; Varga and Heck, 2017; Herrero et al., 2018; Tort et al., 2018; Maric et al., 2020).

Earlier studies revealed that the global signal recorded through functional magnetic resonance imaging (fMRI), which measures changes in blood oxygen level-dependent (BOLD), and is affected by breathing (Birn et al., 2006, 2008a,b). Correcting for breathing signals, for example, significantly improved the detection of the task-related activation (Birn et al., 2006). Later studies, however, recognized the breath as a fundamental rhythm of brain function (Heck et al., 2017). A growing body of literature shows that respiration specifically modulates high frequency oscillations such as gamma activity (40–150 Hz), which are known to govern attention, memory, decision making, problem solving, language processing, and sensory perception (Heck et al., 2017; Kluger and Gross, 2020). In an iEEG study, Herrero et al. (2018) operationalized a breathing awareness task, where participants were asked to count their breaths. In another task, they were instructed to modulate their respiratory rhythm by increasing its pace (Herrero et al., 2018). While gamma band oscillatory activity was shown to correlate with respiratory phase in both the awareness and the modulation of breath, the brain circuits recruited during these tasks did not overlap completely (Herrero et al., 2018). During volitional upregulation of the breathing rhythm, iEEG-breath coherence increases in a frontotemporal-insular network, while during breath counting, activity increased in the ACC, AIC, premotor cortex, and the hippocampus (Herrero et al., 2018). The presence of breathing-entrained gamma band waves across regions in the whole brain suggests that breathing can act as an organizing hierarchical principle for neuronal oscillations (Herrero et al., 2018). Recent studies on respiratory-brain circuitry support this idea (Ito et al., 2014; Biskamp et al., 2017; Heck et al., 2017; Herrero et al., 2018; Tort et al., 2018; Maric et al., 2020).

The entrainment of both slow and fast neural oscillations has been shown to enable neuronal coherence (e.g., Fries, 2005; Lakatos et al., 2005). Breathing has been suggested to exert its system-wide effects in an analogous manner: the slower respiratory rhythm is coupled to faster cortical rhythms which facilitate long distance brain synchronization (Sanchez-Vives and McCormick, 2000; Biskamp et al., 2017; Herrero et al., 2018; Tort et al., 2018; Noble and Hochman, 2019). Both direct and indirect pathways are likely to mediate the process, modulating cortical activity in distinct ways. The rate, phase and type of breathing are all factors which should be considered when discussing the mechanisms underlying respiratory-cortical interactions (for review Maric et al., 2020). Whereas it has been mostly shown that broadband gamma activity couples with respiratory cycle in mice and in humans during normal to fast breathing (Ito et al., 2014; Yanovsky et al., 2014; Zelano et al., 2016; Herrero et al., 2018), others have reported modulation of cortical phase activity in the alpha range across widespread brain areas during slow-paced breathing (Hsu et al., 2020). Overall cross-frequency dynamics involved in controlled breathing, and the factors which influence them, are yet to be delineated. It is nonetheless plausible that the interplay of slow and fast cortical rhythms may be reflecting the relay of bottom-up sensory information and top-down predictions. The reorganization of cortical phase activity may involve an increase in gamma-band coherence and a decrease in alpha-band coherence during the different phases of inspiration. In other words, a hierarchy of neural rhythms may be orchestrating brain-body interactions through an active inference mechanism which mediates information between primary afferent pathways and higher cortical regions (Corcoran et al., 2018). Further, while the work by Tort et al. (2018) disassociates theta and respiration-modulated oscillations, there is evidence that theta and respiration-entrained rhythms are coupled (e.g., Hammer et al., 2020). Interestingly, it seems that they are coupled not through the phase of respiration, but through the rate of breathing (Hammer et al., 2020). Either way, it is likely that respiratory information from pulmonary and nasal pathways is propagated up to cortical areas responsible for the integration of interoceptive and exteroceptive signals (AIC, ACC), and vice versa: top-down predictions issued by cortical regions then regulate physiological responses. The connection between primary sensory regions and the cortex further engages areas associated with emotion (central nucleus of the amygdala, CeA), memory (hippocampus, HC), arousal, and attention (LC). Consequently, modulating the breath means modulating this circuitry, and the physiological responses associated with it. Conceptualized this way, controlled breathing can be framed as an active inference mechanism to regulate a wide array of allostatic processes that together ensure the adaptivity of an organism in a precarious environment.

In this review we choose to focus on respiration-entrained gamma-band neural activity due to their multifaceted roles in mediating physiological, affective cognitive processes (Engel et al., 1999; Heck et al., 2017) and them being likely markers of PE propagation (van Pelt et al., 2016; Boroujeni et al., 2020). It should be noted that this narrowing down is a simplification of the complex rhythmic interactions taking place in a wide network of brain areas, the most relevant of which will be discussed in the next section of this article.

### A Wide Interoceptive Network

According to the current understanding of how the free-energy principle is related to neural oscillations, ascending PEs are conveyed at a faster frequency (e.g., gamma), while descending predictions propagate by lower frequencies (e.g., alpha/beta) (van Pelt et al., 2016; Alamia and VanRullen, 2019). If attention is paid to a sensory signal, in our case the breath, the information it carries in the form of a PE is deemed precise and important. Hence, the weighted PE could propagate to upper levels in the hierarchy, increasing its impact on the updating of high-level predictions (Alamia and VanRullen, 2019). In terms of neural oscillations, this would be reflected by the predominance of feedforward signals, more specifically, gamma-band oscillations propagating from sensory to cortical brain regions. Breathing entrains gamma-band neural oscillatory activity across the brain, which are in turn associated with cognitive and affective functioning (Herrmann et al., 2010; Herrero et al., 2018).

The presence of gamma-band neural oscillations in the AIC is especially interesting in the context of relating the breath to a system-wide regulatory mechanism. For one, this region has been shown to be central in interoceptive attention. In a study by Wang et al. (2019), interoceptive attention was engaged via a breath detection task in which participants were asked to indicate whether a presented breathing curve is delayed or not relative to their own breathing. In another task that engaged exteroceptive attention, participants indicated whether a visual stimulus flashed on the breathing curve. They found that activations in the AIC are critical for interoceptive attention and that they are indicative of individual differences in interoceptive accuracy.

Other studies using breathing tasks to recruit interoceptive attention showed that although interoceptive and exteroceptive attention arise from anatomically distinct regions, they meet in AIC (Farb et al., 2013b,a; Wang et al., 2019; Weng et al., 2020). These findings support already existing interoceptive inference models, which identify the AIC as a region critical for the processing of both interoceptive and exteroceptive signals (Seth et al., 2012; Allen et al., 2019; Allen, 2020). Through its connections to a wider network in brain regions, such as other viscero-motor areas (VMAs), the anterior cingulate cortex (ACC), subgenual cortex (SGC), and orbitofrontal cortex (OCC), the insular cortex can propagate predictions that at the lowest levels serve as homeostatic setpoints, maintained through descending projections to subcortical, brainstem and spinal cord regions. At higher levels, these moderate affective and cognitive states by processing ascending PEs via viscerosensory innervation (Seth and Friston, 2016).

The dynamic coordination of brain activity and physiology orchestrated by interoceptive predictions all works in favor of allostasis (Barrett and Simmons, 2015). Given that respiratory related signaling is a thread linking these pathways, it is plausible that controlled breathing can be used to modulate the processes of physiological, emotional, and cognitive attunement needed to meet ever-changing environmental requirements. More specifically, deliberate modulation of respiration can be used to minimize prediction errors on various levels maximizing the accuracy of interoceptive models, which would result in system-wide changes in the physiological, affective, and cognitive domains.

### Respiration-Specific Circuitry

While an exhaustive overview of respiratory neural circuitry is beyond the scope of the article (for a review Mironov, 2009; Maric et al., 2020), here, we will point out the key players involved in respiratory-entrained cognitive and emotional processes.

These processes, however, cannot be disentangled from the main function of breathing: gas exchange. The lungs, cardiovascular and neural systems work together to supply oxygen (O_2_) to all cells of the body and dispel carbon dioxide (CO_2_). The variations in CO_2_ levels are a potent driver for breathing. Certain neurons in the brainstem, most notably the retrotrapezoid nucleus neurons and serotonergic nuclei, are sensitive to the partial pressure of CO_2_ (pCO_2_) reflecting the amount of CO_2_ dissolved in the blood (Guyenet and Bayliss, 2015). The direct activation of these chemoreceptors is a part of the medullary brainstem circuits involved in the motor control of respiration (Guyenet and Bayliss, 2015). The fluctuations in arterial CO_2_ are also reflected in the global neuronal rhythmicity measured by the fMRI (Wise et al., 2004) and the MEG (Driver et al., 2016). It has been shown that the natural variations in arterial CO_2_ occurring during breathing affect neural oscillations in multiple frequency bands (Driver et al., 2016). These findings suggest that breath-related rhythms have widespread effects on brain activity, the implications of which are yet to be fully understood. A good starting point, however, would be to examine the neuroanatomical connections which link the chemosensing neurons in the brainstem to the rest of the central nervous system.

It has been widely accepted that three parts of the medulla oblongata - the pre-Bötzinger cellular complex (preBötzC), the post-inspiratory complex (PiCo, and the parafacial respiratory group are coupled to orchestrate the timing of inspiration, post-inspiration pause, and expiration, respectively (Maric et al., 2020). Recent work, however, sheds light on the lack of evidence for this triple-oscillatory hypothesis, and the role of the PiCo specifically (Toor et al., 2019; Dhingra et al., 2021). Nonetheless, it is known that the preBötzC sends autonomous action potentials which serve as neural pacemakers (Smith et al., 1991; Del Negro et al., 2018). What makes medulla oblongata especially intriguing is its association with the locus coeruleus noradrenergic (LC/NE) system. The LC/NE system provides the cortex, hippocampus and amygdala with noradrenaline (NE) – a neurotransmitter associated with the modulation of wakefulness, as well as processing of salient sensory information through cognitive operations (Berridge and Waterhouse, 2003). In a recent study, Yackle et al. (2017) identified a cluster of pre-Bötzinger neurons which project to the LC. They propose that these neurons may facilitate a connection between the respiratory center and higher brain regions. Functionally, this may explain how fast breathing in humans leads to an increased state of alertness and arousal (Magalhães et al., 2018). It is worthwhile noting that there are alternative neural circuits which contribute to the voluntary modulation of breathing. Motor cortical pathways involved in volitional respiratory control have long been suggested to bypass the respiratory center and instead act directly on phrenic nerves controlling the diaphragm and consequently respiration (Corfield et al., 1998). More recent anatomical studies reveal a dense reciprocal connectivity between the PAG and the pontomedullary respiratory network in rats (Trevizan-Baú et al., 2021). It is interesting to note that Trevizan-Baú et al. (2021) identify the subparabrachial nucleus as being most densely connected to the PAG, and not the preBötzC, which goes against the idea that corticospinal pathways mediate the volitional control of breathing and instead shows that cortical regions have direct descending pathways connecting them to respiratory control areas. What is also relevant is that the insula provides an equally dense network of descending inputs to the investigated respiratory nuclei (Trevizan-Baú et al., 2021). These findings show that it is not only the breath which affects the cortex, but that the cortex also communicates with the respiratory network – the flow of ascending and descending information has an anatomical substrate. The connection to the PAG is further supported by animal and human studies which link the area to the integration of threat perception with breathing control mechanisms (for review Faull et al., 2015). The nucleus tractus solitarius (NTS), the lateral parabrachial nucleus, and the hypothalamus are in direct communication with the PAG, facilitating processes involved in the control of respiration (Huang et al., 2000; Hayward et al., 2003; Horiuchi et al., 2009). The NTS, specifically, is proposed to act as an important center for cardiorespiratory regulation (Huang et al., 2000; Green et al., 2007). Interestingly, areas implicated in affective evaluation, like the PFC and the amygdala, have been identified to provide input to the PAG (Holstege, 2014). Such studies neuroanatomically support the hypothesis that breathing patterns regulating emotional and allostatic processes may be orchestrated by a respiratory network.

In humans, fMRI studies have indicated which brain regions are affected by the fluctuations of the respiratory rhythm (e.g., Evans et al., 2002; Birn et al., 2008a; Pattinson et al., 2009; Faull et al., 2015). For example, Pattinson et al. (2009) demonstrated that opioid analgesia is related to respiratory neurocircuits. The authors showed that the urge to breathe activates the secondary somatosensory cortex, the frontal operculum and the bilateral insula. An opioid, remifentanil, reduced the awareness of respiration and led to a decrease in BOLD response in the PAG, the cerebellum, ACC, and the dorsolateral PFC. Holding the breath was associated with activations in the brainstem, and more concretely the medulla oblongata. Overall, both brainstem and higher cortical regions seem to be crucial players in facilitating the awareness of breathing necessary for its volitional modulation. Although future neuroanatomical studies should identify the exact circuitry that governs the interactions between respiration, neural oscillations and the free energy principle, evidence suggests that the network of brain regions modulated by respiration affect a wide range of processes such as attention, emotion, and memory through bidirectional processing of bottom-up and top-down signals.

**FIGURE 1:**
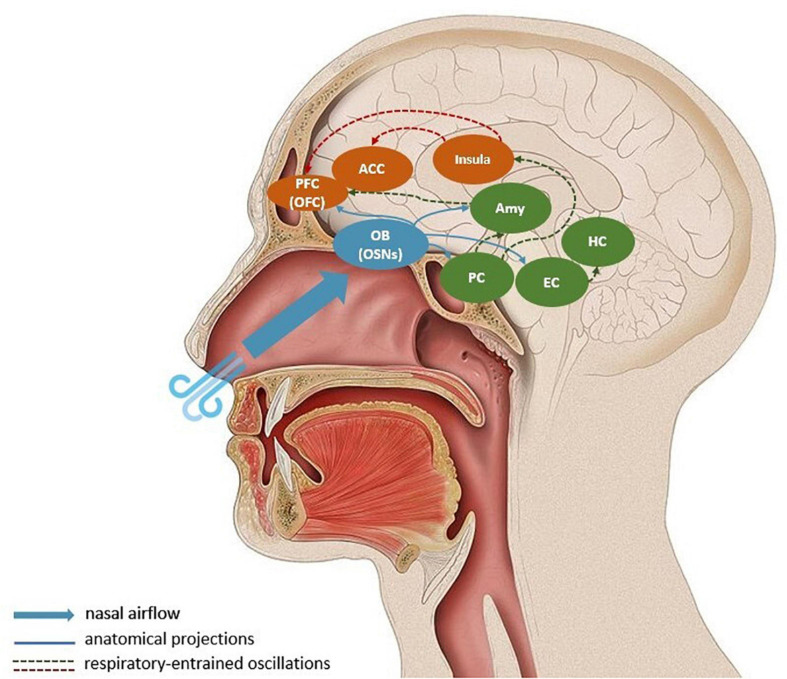
Respiratory-entrained propagation of prediction errors. Nasal breathing stimulates olfactory sensory neurons in the olfactory bulb, which synchronizes respiratory rate and neural oscillations in the olfactory bulb and piriform cortex. A large-scale entrainment of downstream targets like the entorhinal cortex, hippocampus, the amygdala, insula, orbitofrontal cortex, and anterior cingulate cortex is suggested to co-occur. During controlled respiration prediction errors, or bottom-up sensory information, are propagated to visceromotor corical regions (mostly via the insula) where they can update generative models. The blue arrows represent direct anatomical projections from the olfactory bulb; the dashed arrows indicate long-distance respiratory-entrainment, where the red lines show the relay of prediction errors in the form of gamma-band oscillations. For simplification, the descending propagation of predictions from cortical regions to physiological regulatory centers is omitted from the schema. Amy, Amygdala; ACC, anterior cingulate cortex; EC, entorhinal cortex; HC, hippocampus; OFC, orbitofrontal cortex; OB, olfactory bulb; OSNs, olfactory sensory neurons.

In addition to the medullary brainstem circuits outlined above, the respiratory rhythm affects neural activity through the oscillatory entrainment of a large network of brain regions, in which nasal breathing is suggested to play an important role (e.g., Zelano et al., 2016; Herrero et al., 2018). Breathing through the nose seems to be key when discussing how controlled breathing affects motility, emotions, cognition and sensory processing (Zelano et al., 2016; Ma et al., 2017; Wells et al., 2020). A respiration-specific neural mechanism, which relays sensory information through the olfactory bulb (OB) may explain why. Nasal breathing activates olfactory sensory neurons (OSN) in the nasal cavity, which project to the OB (Cunningham et al., 1999). In rodents, OB has been shown to be linked to respiratory-entrained low-frequency oscillations in the barrel cortex (Fontanini and Bower, 2005; Buonviso et al., 2006; Ito et al., 2014). Olfactory and whisker-related information is expected to be synchronized with respiration: sniffing is rhythmically coupled with nasal, head and whisking movements, facilitating active sensing (Verhagen et al., 2007; Corcoran et al., 2018). Here, active sensing expresses the idea that the environment is explored with a purposeful motor strategy to extract relevant sensory information. The synchronized whisking-related activity can support the flow of bottom-up sensory information and top-down predictions. Hence, OB-mediated oscillations related to olfaction can be framed as an active inference strategy to reduce uncertainty and support adaptive action (Corcoran et al., 2018). However, the presence of respiratory-entrained neural oscillations in other brain regions not directly related to olfaction remain puzzling (Yackle et al., 2017; Zhong et al., 2017; Herrero et al., 2018; Tort et al., 2018). As shown by Zelano et al. (2016), nasal, but not mouth, breathing entrain electrical activity in the human olfactory cortex, as well as in limbic brain areas, such as the amygdala and the hippocampus. It is, therefore, likely that the long-range synchrony of neural oscillations in the gamma-band and the cognitive functioning related to it is mediated through slow respiratory-entrained oscillations through OSNs and the OB. In Figure 1, we illustrate the key regions involved in the interaction between breathing, gamma-band oscillations and prediction error propagation.

## Breathing Out: An Integration of the State of the Art and Its Significance

The crux of the significance of controlled respiration is that breathing always happens in the present moment - it grounds the organism in the current sensory experience. In the context of the free-energy principle, this is significant because it implies that the breath can be used to recontextualize bottom-up respiratory-related sensory information, which could alter top-down predictions. For instance, slowing the breathing rhythm triggers a number of physiological, cognitive and affective responses, corresponding to a generative model of an overall relaxed state. Conversely, a rapid breathing rhythm elicits an aroused state. Conscious control of the breath can therefore be used as a fast-acting tool to modify interoceptive priors.

The overarching purpose of this respiration-based regulatory mechanism is to enable adaptation to a changing environment. To recapitulate with an example: in an anxiety-provoking situation, one can choose to attend to the breath and slow it down, and by doing so alter an anxious internal state to a relaxed one, without having changed environmental circumstances. If the same external states demand different optimal reactions in different situations, through this mechanism, the organism can react flexibly. In predictive processing terms, uncertainty is minimized by putting more weight on bottom-up respiratory signals and modulating predictive interoceptive models through controlled breathing. Thousands of years of breathing practices are thus grounded in an integration of contemporary findings in the fields of respiratory neurophysiology, psychology, and neuroscience.

### Open Questions and Implications

From the insights discussed in this article, controlled breathing emerges as an active inference strategy for minimizing interoceptive prediction errors. However, controlled breathing is a concept which would benefit from improved granularity, as it remains to be clarified what types of breathing are best suited for which situation, how long-lasting their effects are and if the effects are prolonged by practice. In this paper, we refer to the changes that occur as soon as the breath is brought into conscious awareness and modulated. The time scale on which these changes occur may vary from individual to individual and context to context. Future research should elucidate which factors influence this process. While the effects of slow breathing (from 4–10 breaths per min) on the cardiovascular and autonomic nervous system have been well documented (Russo et al., 2017), a multitude of fast-paced breathing techniques remain to be systematically studied (Saoji et al., 2019). The same holds for the role of nasal breathing, and its variations in terms of nostril constriction, in opposition to mouth breathing, as the neurocircuitry engaged by them does not overlap completely (Maric et al., 2020).

Perhaps more importantly, however, the question of how controlled breathing would act as an active inference mechanism to alter interoceptive predictions should be further clarified and formalized. Animal models utilizing viral tracing techniques are crucial to disentangle the underlying neurocircuitry. Once established, the connectivity can be causally analyzed using optogenetics to selectively target and manipulate neuronal networks. In human neuroimaging studies, a combination of EEG, fMRI, and respiratory measurements can be used to investigate how the breath entrains cortical regions and whether the OB-AIC-Frontal cortex circuitry should be the focus of future investigation.

Another standing question is the significance of *perceptual inference* within the discussed processes. This issue is tightly connected to the role of attention within the free-energy principle. While heightened weighting of sensory evidence mediated by attention attenuates predictions and increases the precision of *perceptual inference* (Kok et al., 2012), it is unclear whether attention *per se* is enough to elicit a change in the interoceptive models. Put differently, it should be further discussed whether paying attention to a certain feature of the external or internal environment can be considered an “action” by itself, transcending the function of precision weighting (Clark, 2017).

Underlying this proposal is the motivation to contribute to the physiological and the psychological well-being of both clinical and normative populations. Maintaining a state of inner balance within an ever-changing environment is a process which requires receptivity and flexibility. In the vast majority of time, attuning to the internal and external environment does not require conscious awareness. In fact, high interoceptive awareness is associated with anxiety in both healthy subjects and in clinical populations (Zoellner and Craske, 1999; Critchley et al., 2004; Eley et al., 2004). However, given the promising results of interoceptive training on the reduction of anxiety (Garfinkel et al., 2017; Sugawara et al., 2020), it is likely that this not an issue of an increased interoceptive sensitivity *per se*. On a psychological level, the training may be effective because it teaches a different mode of relating to these signals, which on a computational level can be framed as a strategic weighting of sensory evidence. This is in line with the already well-established importance of interoceptive signal processing in emotional stability and mental health (Barrett et al., 2016; Critchley and Garfinkel, 2017; Murphy et al., 2017; Khalsa et al., 2018). Therefore, controlled breathing has the potential to be established as an easily accessible and effective tool, which shapes emotional, cognitive, and physiological processes.

### Conclusion

This article integrates respiration, neural oscillations and the free-energy principle into a unified regulatory network of interaction between physiological, affective, and cognitive processes. Much like an inhale gives rise to an exhale, the conceptual framework can give rise to research. For this research to be exhaustive and to do justice to the complexity of the interactions taking place, it requires experts from different fields to combine their skills and share their knowledge. Computational models and empirical studies ought to establish how respiration entrains the central nervous system and how this is tied to the homeostatic balance of the whole organism. Based on the interactions outlined here, we believe that investigating how gamma oscillations, via the OB, entrain processes in the insula is a promising direction to go into, which would help to understand how neuronal oscillations at different temporal scales fit within the free-energy principle. This would be the starting point for establishing scientifically sound strategies to modulate maladaptive interoceptive models, as those associated with anxiety, alexithymia, ADHD, depression and eating disorders (Seth and Friston, 2016). An interdisciplinary approach would be vital in deepening the understanding of how the brain, body and the environment interact.

## Author Contributions

AB and EK contributed to the conceptualization, writing, and revision of the manuscript. AB conceived the original idea. EK closely supervised the project. Both authors contributed to the article and approved the submitted version.

## Conflict of Interest

The authors declare that the research was conducted in the absence of any commercial or financial relationships that could be construed as a potential conflict of interest.
